# Alcohol Tax Policy and Related Mortality. An Age-Period-Cohort Analysis of a Rapidly Developed Chinese Population, 1981–2010

**DOI:** 10.1371/journal.pone.0099906

**Published:** 2014-08-25

**Authors:** Roger Y. Chung, Jean H. Kim, Benjamin H. Yip, Samuel Y. S. Wong, Martin C. S. Wong, Vincent C. H. Chung, Sian M. Griffiths

**Affiliations:** Jockey Club School of Public Health and Primary Care, Faculty of Medicine, The Chinese University of Hong Kong, Hong Kong SAR, The People's Republic of China; National Taiwan University, Taiwan

## Abstract

To delineate the temporal dynamics between alcohol tax policy changes and related health outcomes, this study examined the age, period and cohort effects on alcohol-related mortality in relation to changes in government alcohol policies. We used the age-period-cohort modeling to analyze retrospective mortality data over 30 years from 1981 to 2010 in a rapidly developed Chinese population, Hong Kong. Alcohol-related mortality from 1) chronic causes, 2) acute causes, 3) all (chronic+acute) causes and 4) causes 100% attributable to alcohol, as defined according to the Alcohol-Related Disease Impact (ARDI) criteria developed by the US Centers for Disease Control and Prevention, were examined. The findings illustrated the possible effects of alcohol policy changes on adult alcohol-related mortality. The age-standardized mortality trends were generally in decline, with fluctuations that coincided with the timing of the alcohol policy changes. The age-period-cohort analyses demonstrated possible temporal dynamics between alcohol policy changes and alcohol-related mortality through the period effects, and also generational impact of alcohol policy changes through the cohort effects. Based on the illustrated association between the dramatic increase of alcohol imports in the mid-1980s and the increased alcohol-related mortality risk of the generations coming of age of majority at that time, attention should be paid to generations coming of drinking age during the 2007–2008 duty reduction.

## Introduction

It has long been shown that government alcohol policies such as taxation directly influence the quantity of alcoholic beverages imported and sold in a country, which, in turn, correlate strongly with population alcohol consumption levels. A meta-analysis revealed that a decrease of 10% in prices results in about 5% increase in consumption [1], and most studies have demonstrated an association between high alcohol consumption and poorer health outcomes [2]–[5].

Previous studies have demonstrated the importance of considering the three time-related effects of age, period and cohort when examining the trends in alcohol consumption [6]–[10]; however, the influence of these effects on the trends of alcohol-related health outcomes have been understudied [11], [12]. Moreover, less is known about the role of these effects in influencing alcohol-related health outcomes in a population that is traditionally not known to have high alcohol consumption levels.

**Figure 1:**
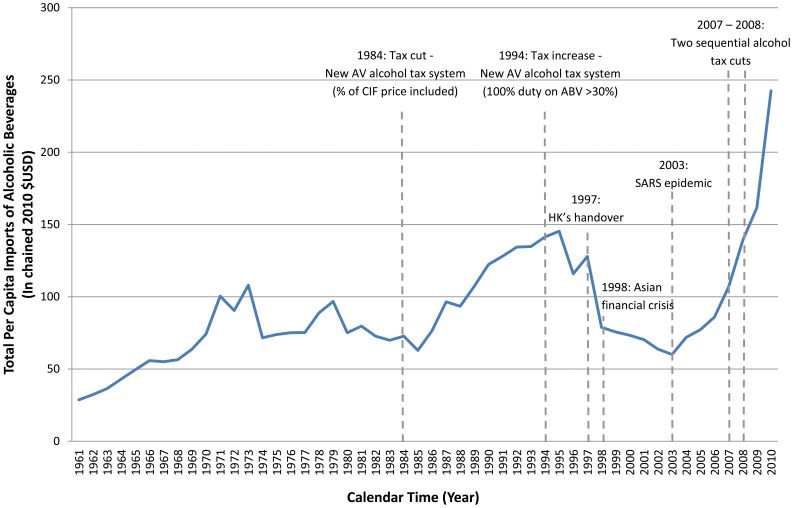
Total per capita imports of alcoholic beverages ($ at current market prices) for Hong Kong population aged 20 or above, 1961–2010.

Hong Kong has experienced rapid socio-economic transition from pre-industrial to post-industrial living conditions over the past few decades. In parallel, several important alcohol tax policy changes were implemented. Historically known to be a ‘duty-free’ port, imports on alcoholic beverages have been subject to special excise taxes since 1909 [13]. Three major alcohol tax policy changes have since taken place that significantly influenced the quantity of alcoholic beverages imported into Hong Kong. Figure 1 illustrates the trend of the total *per capita* imported dollar value (in real term $USD) of alcoholic beverages in relation to the alcohol tax policy changes over time in Hong Kong. Specifically, the 1984 tax cut was followed by an increase in total *per capita* imports of alcoholic beverages [13], while the 1994 tax increase was followed by a decrease in total *per capita* imports [14]. In order to increase revenues from the hospitality industry, to capitalize on greater tourism and to promote Hong Kong as Asia's foremost wine hub [15], [16], an unprecedented sequential change in alcohol tax policy occurred in 2007 and 2008 when alcohol tax was reduced and altogether eliminated, respectively. These two successive large-scale duty reductions resulted in an exponential growth of alcohol imports. Although a recent time-series study [17] suggested an association between the 2007 alcohol duty reduction and increased cardiovascular mortality in Hong Kong from 2001 to 2010, no Chinese studies have ever simultaneously modeled the three time-related factors of age, period and cohort, which is necessary to delineate the temporal dynamics between alcohol tax policy changes and related health outcomes. Given that the social drinking trends in the West differ from that observed in Hong Kong Chinese society where there is generally less heavy drinking [18]–[20], implications can be particularly telling.

Taking advantage of the natural experiment provided by Hong Kong's alcohol policy history, and the fact that Hong Kong has had limited commercial production of alcohol locally [21], we conducted the first age-period-cohort (APC) model of the alcohol-related mortality in any Chinese population to delineate the possible impact of age, calendar period and birth cohort on alcohol-related health outcomes. In APC models, age is a surrogate of the aging process and represents cumulative exposures over an individual's life course. Period effects are population-wide exposures which affect individuals of all ages during a certain period (e.g. historical events, economic downturns and changes in government policies) [22]. They demonstrate the most explicit influence on alcohol consumption levels [7]. Last, the cohort effect is a surrogate of the effects of factors related to the time of birth, because the same birth cohorts may have the same exposures at various critical periods of their lives [22]–[24]. Alcohol policy changes may have varying effects on different birth cohorts in the same underlying population, since it has been shown that younger people are more easily influenced by their environment [25]. In this study, we hypothesize that the period effect of alcohol-related mortality would correlate strongly with changes in imports of alcoholic beverages. Also, since the post-1960's Hong Kong birth cohorts would be the first to have reached their age of majority when mass amounts of alcohol became commercially available in the mid-to-late 1980s due to the tax cut in 1984, we thereby hypothesize that these individuals would possess an increased mortality risk from alcohol-related causes.

## Methods

### Data sources

The Hong Kong Census and Statistics Department provided mid-year population figures and all known (i.e., registered) deaths in Hong Kong from 1980 to 2010 by age, sex and cause of death. Causes of death were coded using the Ninth Revision of *International Classification of Diseases* (ICD-9) for 1980–2000, the ICD-10 for 2001 onwards.

### Outcomes

Alcohol-related mortality is defined according to the Alcohol-Related Disease Impact (ARDI) criteria developed by the US Centers for Disease Control and Prevention (CDC) [26], where all alcohol-related causes are categorized into chronic and acute causes. They are further categorized according to their estimated direct and indirect alcohol-attributable fractions. Please refer to Table S1 and S2 for the respective lists of the chronic and acute alcohol-related causes by alcohol-attributable fractions and their corresponding ICD-9 and ICD-10 codes. The estimated number of deaths attributable to alcohol for each cause was calculated by multiplying the actual counts of death for each cause by the corresponding alcohol-attributable fraction.

Specifically, we considered the following types of alcohol-related mortality: 1) chronic-cause alcohol-related mortality (chronic-ARM), 2) acute-cause alcohol-related mortality (acute-ARM) and 3) all-cause (chronic and acute) alcohol-related mortality (all-ARM). For comparison, we also considered all-cause alcohol-related mortality that were 100% attributable to alcohol (100% attributable-ARM), which is how most other studies in the literature defined alcohol-related causes.

### Data analysis

Mortality rates for the population were expressed per 100 000 people and directly standardized to the World Standard Population [27]. We used quinquennial age groups (ranging from 20–24 to 85+ years) over six quinquennial periods (from 1981 to 2010). The 19 birth cohorts ranged from 1894 to 1988.

We applied sex-specific Poisson APC models using maximum likelihood method to estimate the relative risks by age, period, and cohort, with 95% confidence intervals. Details of the model are specified in Note S1. APC models identify broad or macro-environmental risk factors that may contribute to changes in disease patterns by decomposing mortality rates by age, period and cohort effects [28], [29].

Our approach to dealing with the non-identifiability problem of age, period and cohort effects [30] was to adopt the commonly used technique of imposing an additional arbitrary reference constraint for the period effect [31]–[35]. In particular, we constrained the second and penultimate periods to be reference categories [33]–[35]. Due to the non-identifiability problem, only second-order changes (i.e. inflection points in the slope) of age, period and cohort effects can be interpreted. We plotted the estimates of these three effects to facilitate the visualization of second-order changes. Our models used the Akaike Information Criterion (AIC) to evaluate the contribution of age, period and cohort. A lower AIC indicates a better fitting model, and hence a significant change in effect through time for the relevant component. All analyses were implemented in R version 2.5.0 (R Development Core Team, Vienna, Austria).

## Results

### Age-standardized mortality rates

**Figure 2:**
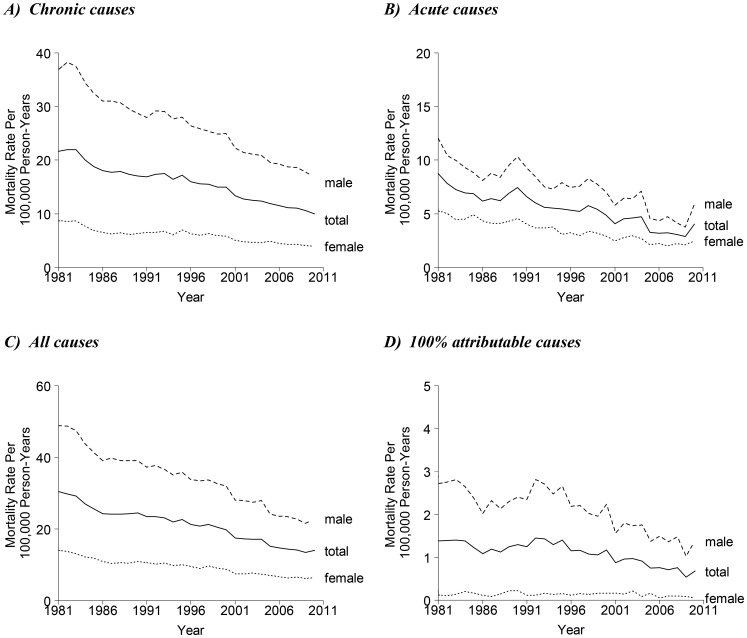
Age-standardized alcohol-related mortality rates in Hong Kong, 1981–2010, among men (dashed line), women (dotted line) and both sexes (solid line) for A) chronic causes, B) acute causes, C) all (chronic and acute) causes, and D) 100% attributable causes.

Figure 2 shows the observed age-standardized mortality rates for chronic-ARM, acute-ARM, all-ARM from 1981 to 2010 in Hong Kong. Trends for 100% attributable-ARM are also shown for comparison. For each of these mortality rates, there was a decline for both sexes across time. The decline was less marked for 100% attributable-ARM among females, whose mortality rate had remained low throughout the years. Moreover, dramatic increases of mortality rates in acute-ARM and 100% attributable-ARM were observed in males in 1986–1990 (following the 1984 cut of the alcohol duties) and in 2009–2010 (following the eradication of the wine and beer duties). The corresponding increase was less apparent in chronic-ARM.

### Age, period and cohort effects

**Figure 3:**
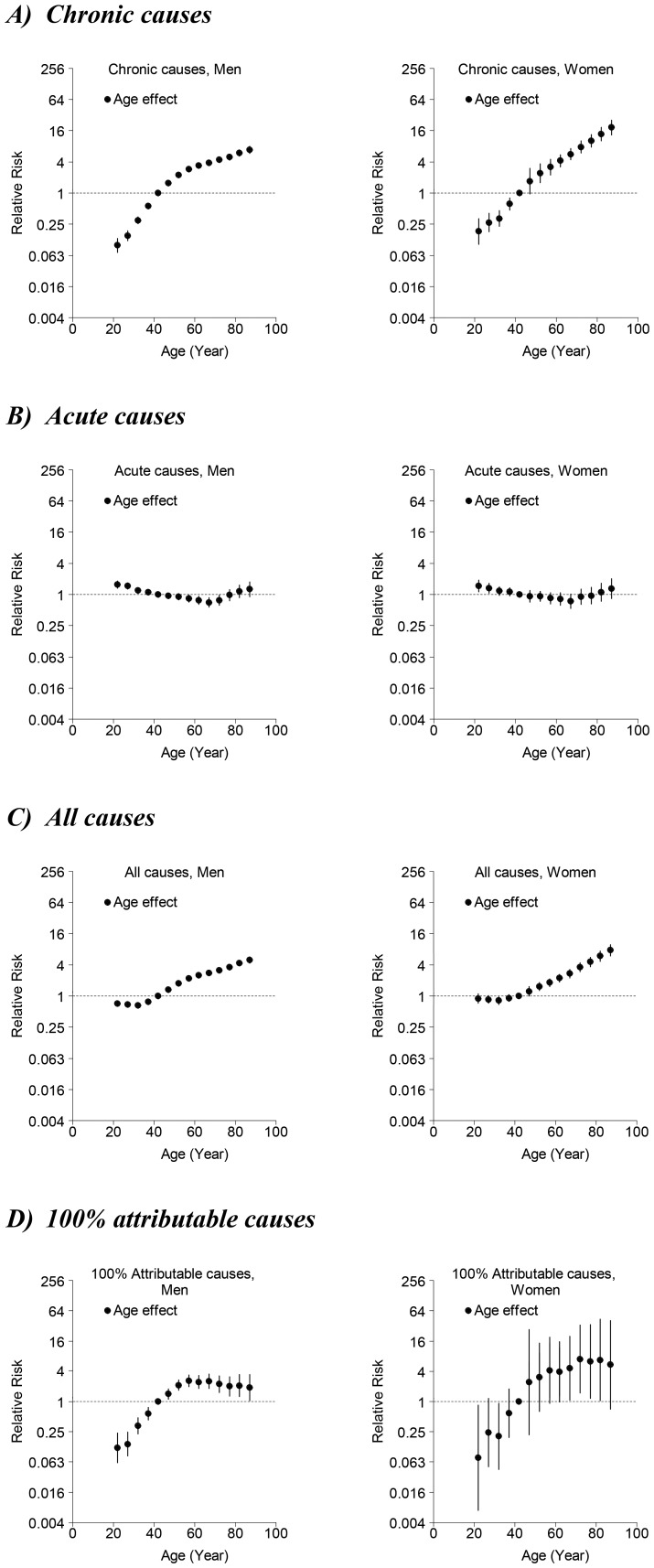
Parameter estimates and 95% confidence intervals of age effect of alcohol-related mortality among men (left-hand panels) and women (right-hand panels) for A) chronic causes, B) acute causes, C) all (chronic and acute) causes, and D) 100% attributable causes in Hong Kong, 1976–2010.

**Figure 4:**
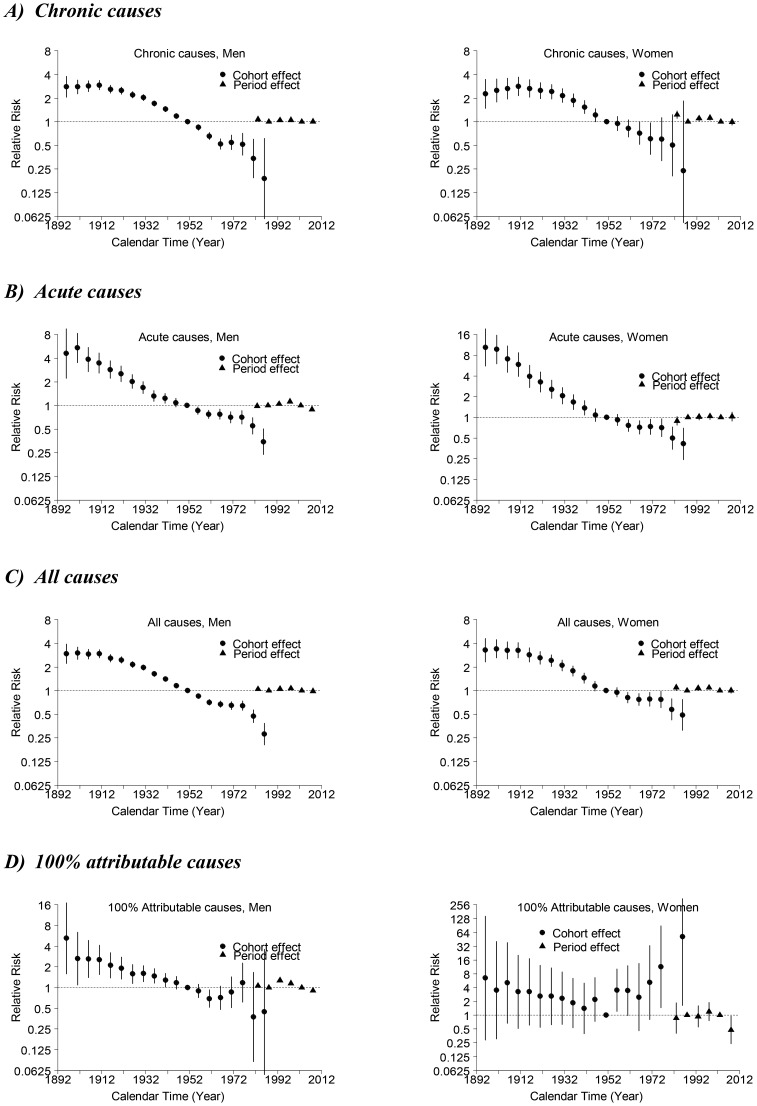
Parameter estimates and 95% confidence intervals of period (triangles) and cohort (circles) effects of alcohol-related mortality among men (left-hand panels) and women (right-hand panels) for A) chronic causes, B) acute causes, C) all (chronic and acute) causes, and D) 100% attributable causes in Hong Kong, 1976–2010.

For almost all models, age, period and birth cohort contributed to the observed changes in mortality. In general, models including all three components fitted best. Figure 3 (Figure 4) shows the relative risks by age (period and cohort), from which second-order changes have been identified. In general, mortality increased with age and decreased with younger birth cohort.

#### Age effects (Figure 3)

All-ARM increased with age. There were upward inflections after 30–34 year-old for both sexes. In males this was followed by a slight downward inflection after 55–59 year-old. Although chronic-ARM increased with age; there was a decrease in mortality risk for men after 50–54 year-old and for women after 55–59 year-old. Acute-ARM, however, had an upward inflection for both sexes after 65–69 year-old. 100% attributable-ARM increased with age for both sexes, until the age of 55–59 years where there was a downward inflection.

#### Period effects (Figure 4)

In terms of period effects for both sexes in chronic-ARM and all-ARM, there was a slight upward inflection after 1986–1990, a slight downward inflection after 1996–2000, followed by an upward inflection after 2001–2005. For acute-ARM, there was an upward inflection around 1986–1990 and a downward inflection around 1996–2000 for men, whereas for women, the trend was less apparent. For 100% attributable-ARM, there was an upward inflection after 1986–1990 followed by a downward inflection after 1991–1995 for men. Due to relatively small number of deaths, the confidence intervals were wide and the pattern was unapparent for women.

#### Cohort effects (Figure 4)

In terms of cohort effects, there was a downward inflection for both sexes born around the 1910s for all-ARM and chronic-ARM, but not for acute-ARM and 100% attributable-ARM. For both sexes in acute-ARM and all-ARM, there was an upward inflection for both sexes born around the late-1950s to early-1960s, followed by a downward inflection around mid-1970s. For chronic-ARM, there was an upward inflection around mid-1960s followed by a downward inflection around mid-1970s for both sexes, but the upward inflection was more apparent for men. For 100% alcohol-attributable mortality, the pattern was even more unapparent for both sexes with very wide confidence intervals due to relatively small number of deaths.

## Discussion

Our findings illustrated possible effects of macro-environmental events (i.e., alcohol policy changes and major socio-economic events) on alcohol-related mortality. The age-standardized mortality trends were generally in decline, with some apparent fluctuations coinciding with the occurrence of alcohol policy changes. On the other hand, the APC analyses suggested possible temporal dynamics between alcohol policy changes and alcohol-related mortality through the period effects and generational impact through the cohort effects. Moreover, we compared the APC results of alcohol-related diseases to other non-alcohol-related diseases, which serve as “control” outcomes to identify systematic changes. In particular, we respectively compared chronic alcohol-related mortality with lung cancer mortality, and acute alcohol-related mortality with respiratory disease mortality, as observed in a previous Hong Kong study [33]. No similarities in terms of the patterns and changes of slope of the effects were observed, implying that the results in the present study were not merely reflections of systematic changes.

### Limitations

We should interpret our findings with caution. First, Since APC modeling is an ecological design and is descriptive in nature, we can only speculate about the etiologies of the observed changes. This is an inherent limitation to the APC modeling. Nevertheless, APC models are particularly valuable in generating hypothesis and providing insights into the possible etiologic and macro-environmental factors associated with the temporal variations of the disease trend that may otherwise be overlooked. Also, they are especially useful in recently developing and developed locations, where long-term records or cohort studies may be lacking. Second, the findings' validity also depends on the quality of the mortality and population data. Nonetheless, the majority of deaths in Hong Kong have taken place in hospitals, especially since the 1970s, facilitating accurate ascertainment of causes of death. Also, as in many developed countries, autopsy rates have declined and some misdiagnoses are inevitable [36], but there is no reason to believe that such misdiagnoses should be systematic. Although many residents born before the 1960s were post-war migrants who may lack birth certificates, age misclassifications, if any, should be minor and likely be reduced by the quinquennial age grouping in the data analyses.

### Temporal impacts of alcohol policy changes

#### Impact of 1984 alcohol tax cut

After the 1984 tax cut, there was an increase of age-standardized acute-ARM among men from 1986 to 1990, which coincided with the surge of alcohol imports during the same time period (Figure 1). While the two events coincided temporally, it is still uncertain whether this increase of mortality over the four-year period was due entirely to the tax cut. Other macro-environmental changes, such as increased attention by medical professionals and increased overall death registration, could also account for the increase in age-standardized acute-ARM. Nevertheless, it is difficult to argue why attention by medical professionals would fluctuate to a degree to macro-environmentally influence the mortality rate of the population for four years only but not afterwards. Also, no general increase of age-standardized all-cause mortality was observed during 1986 to 1990 in Hong Kong [33].

On the other hand, the increase was a lot less apparent for chronic-ARM, which is reasonable given that it usually takes longer time for drinkers to develop and die from chronic causes. Such increases were even less apparent among women, probably because they generally drink less alcohol than men [37], [38]. However, increase in chronic-ARM also did not happen 20 to 30 years after the 1984 tax cut. This was plausible because too many macro-environmental factors could have contributed to changes of the general mortality trend. On the contrary, such long-term impact would be more easily detectable in the form of cohort effect which can identify changes in the risk of mortality among each specific birth cohort who had similar generational exposures. We would discuss more on the generational impact of alcohol policy changes later in the corresponding section of the Discussion.

Similar to observations made from the age-standardized mortality trend, our period effect showed an upward inflection in risk of mortality among men for acute-ARM after 1986–1990, following the surge of alcoholic imports during mid-1980s. Conversely, no second-order increase was observed for the period effect of acute causes among women, possibly because women tended to drink less than men, giving lower numbers of acute deaths in general. Moreover, an apparent second-order increase of period effect was observed for chronic-ARM and all-ARM in both sexes during late 1980s. While this increased period effect seems to contradict to the generally declining age-standardized mortality rate, the slight increase of age-standardized mortality rate during late 1980s may imply that chronic-ARM and all-ARM could have possibly seen a faster decline if no alcohol policy changes occurred in 1984.

#### Impact of 1994 alcohol tax increase

Second-order decrease in risk of mortality was universally observed in the period effect for chronic, acute and all-ARM for both sexes after 1996–2000. The decrease in risk coincided with the decline in imports of alcoholic beverages. After the change in taxation policy in 1994, imports started to decrease in 1995, with a tentative recovery in 1997 possibly linked to the celebration of the handover of Hong Kong to China, and continued to decrease afterwards.

#### Impact of 2007 and 2008 alcohol tax cuts

The increase in imports that happened after the two consecutive tax cuts in 2007 and 2008 was the greatest ever in Hong Kong's history (Figure 1). The increase in age-standardized mortality rate for acute causes from 2009 to 2010 among men might be linked to the tax cuts, and this parallels the findings of another recent Hong Kong study [39] showing that people ever drinking alcohol significantly increased and people with lower educational achievement and the unemployed are more likely to binge drink after the tax cut. Nevertheless, since these tax cuts were only recent events, the observed increase might well reflect a natural variability of the mortality trend. A longer series of prospective data is warranted to verify the association.

On the other hand, we observed a second-order increase in the period effect among both sexes after 2001–2005 for chronic-ARMs and all-ARMs. It is possible that the two consecutive tax cuts contributed to the increased risk of dying from alcohol-related chronic causes, and this is consistent with the pattern observed for the period effect during late 1980s after the implementation of the alcohol tax cut in 1984. The lack of apparent second-order increase in risk of acute-ARM among both sexes after 2001–2005 was probably due to the increased mortality rate linked to the Asian financial crisis and SARS epidemic during the early-2000s, thus generating no significant second-order increase in the period effect after 2001–2005.

### Generational impact of alcohol policy changes

While changes in alcohol policy had explicit impacts on the risk of alcohol-related mortality on a population-wide scale (i.e., period effects), they could also adversely affect the health of different generations (i.e., cohort effects). There was consistently an upward inflection of risk of chronic-ARM, acute-ARM and all-ARM for both sexes born around the 1960s. The post-1960s birth cohorts were the first generations in Hong Kong to be exposed to a more developed economic environment and significantly increased alcohol availability on the market as a result of the 1984 alcohol policy changes when they first reached their age of majority. Since younger people tend to be more easily influenced by their surroundings [25], and studies have consistently shown associations of early alcohol use with increased risk of alcohol-related problems later [40], [41] as well as of road traffic accidents for the young new-licensed drivers [42], [43], extra attentions should be paid to the first generations coming of age of majority. In Hong Kong's case, this would be for the 1980s cohorts because they would be the first generation exposed to the second dramatic increase of imports of alcoholic beverages resulting from the two consecutive alcohol tax cuts in 2007 and 2008.

There was also a consistent second-order decrease of risk of chronic-ARM, acute-ARM and all-ARM for both sexes born around the 1970s, possibly due to improvements in public health interventions (e.g. general improvements in medical technology, increasing prevalence of hepatitis B vaccination [44] and more stringent driving safety regulations [45]).

### Different definitions of alcohol-related mortality

The comparison of the findings using the comprehensive definition of alcohol-related causes provided by the CDC ARDI criteria versus using the more common definition of causes 100% attributable to alcohol illustrated the possible underestimated overall risk of alcohol if only causes that are obviously attributable to alcohol are included. For example, diseases such as chronic pancreatitis, liver cirrhosis and liver cancer, were not 100% but fractionally attributable to alcohol consumption. As a consequence, the upward inflection observed around 65–69 years for acute-ARM cannot be captured when only causes 100% attributable to alcohol were considered. On the contrary, a decline in age effect around 55–59 years was observed for 100%-attributable ARM, which may create an illusion that risk for alcohol-related mortality decreased in older age.

Another significant difference between the two definitions was the absence of an upward inflection in the period effect after 2001–2005 for 100% attributable-ARM, but the presence of one using the CDC ARDI criteria. Specifically, the impact of policy changes in 2007 and 2008 on population health may be underestimated when only causes 100% attributable to alcohol were considered. This supports the consideration to adopt the comprehensive definition of alcohol-related causes which included causes 100% attributable and fractionally attributable to alcohol in future studies in the field.

### Conclusion

This is the first study that showed possible temporal association between Hong Kong's changing alcohol policy and alcohol-related mortality by age, period and cohort effects. While reductions of alcohol duties had explicit population-wide impact on the risk of alcohol-related mortality, it could also adversely affect the health of generations being exposed to increased alcohol availability on the market upon reaching their age of majority. Based on our findings, attention should then be paid to generations coming of drinking age during the 2007–2008 duty reduction. Our findings suggested decreasing alcohol duties may have long-term population-level health harms beyond the acute ones typically associated with alcohol consumption. Future studies should follow cohorts longitudinally to scrutinize the effects of alcohol consumption on mortality risk.

## Supporting Information

Table S1 **Chronic alcohol-related causes by AAF and their corresponding ICD-9 and ICD-10 codes.**
(PDF)

Table S2 **Acute alcohol-related causes by AAF and their corresponding ICD-9 and ICD-10 codes.**
(PDF)

Note S1 **Technical Appendix of the Poisson age-period-cohort model.**
(PDF)
