# Supplementary material for: Alcohol Tax Policy and Related Mortality. An Age-Period-Cohort Analysis of a Rapidly Developed Chinese Population, 1981–2010
Source: PLoS One. 2014 Aug 25;9(8):e99906. doi: 10.1371/journal.pone.0099906 (PMC4143164; doi:10.1371/journal.pone.0099906)
Supplement: Table S1 — Chronic alcohol-related causes by AAF and their corresponding ICD-9 and ICD-10 codes. (PDF) [file pone.0099906.s001.pdf]

Table S1. Chronic alcohol-related causes by AAF and their corresponding ICD-9 and ICD-10 codes.

**Cause**	**ICD-9**	**ICD-10**
100% Attributable		
Alcoholic psychosis	291	F10.3-F10.9
Alcohol abuse	305.0, 303.0	F10.0, F10.1
Alcohol dependence syndrome	303.9	F10.2
Alcohol polyneuropathy	357.5	G62.1
Degeneration of nervous system due to alcohol	Nil	G31.2
Alcoholic myopathy	Nil	G72.1
Alcohol cardiomyopathy	425.5	I42.6
Alcoholic gastritis	525.3	K29.2
Alcoholic liver disease	571.0-571.3	K70-K70.4, K70.9
Fetal alcohol syndrome	655.4, 760.71	Q86.0
Fetus and newborn affected by maternal use of alcohol	Nil	P04.3, O35.4
Alcohol-induced chronic pancreatitis	Nil	K86.0
Direct Alcohol-Attributable Fractions Estimate		
Acute pancreatitis	577.0	K85
Chronic pancreatitis	577.1	K86.1
Epilepsy	345	G40, G41
Esophageal varices	456.0-456.2	I85, I98.20, I98.21
Gastroesophageal hemorrhage	530.7	K22.6
Liver cirrhosis, unspecified	571.5-571.9	K74.3-K74.6, K76.0, K76.9
Portal hypertension	572.3	K76.6
Spontaneous abortion	634	O03
Indirect Alcohol-Attributable Fractions Estimates (English et al. and Ridolfo and Stevenson cut points)		
Breast cancer, females	174	C50
Cholelithiases	574	K80
Chronic hepatitis	571.4	K73
Esophageal cancer	150	C15
Hypertension	401-405	I10-I15
Ischemic heart disease	410-414	I20-I25
Laryngeal cancer	161	C32
Liver cancer	155	C22
Low birth weight, prematurity, intrauterine	656.5, 764, 765	O36.5, O36.4, P05, P07

growth retardation or death		
Oropharyngeal cancer	141, 143-146, 148, 149	C01-C06, C09-C10, C12-C14
Psoriasis	696.1	L40.0-L40.4, L40.8, L40.9
Superventricular cardiac dysrhythmia	427.0, 427.2, 427.3	I47.1, I47.9, I48
Indirect Alcohol-Attributable Fractions Estimates (Corrao et al. and Bargnardi et al. cut points)		
Stroke, ischemic	433-435, 437, 362.34	G45, I63, I65-I67, I69.3
Stroke, hemorrhagic	430-432	I60-I62, I69.0-I69.2
Prostate cancer	185	C61
