# Supplementary material for: Alcohol Tax Policy and Related Mortality. An Age-Period-Cohort Analysis of a Rapidly Developed Chinese Population, 1981–2010
Source: PLoS One. 2014 Aug 25;9(8):e99906. doi: 10.1371/journal.pone.0099906 (PMC4143164; doi:10.1371/journal.pone.0099906)
Supplement: Table S2 — Acute alcohol-related causes by AAF and their corresponding ICD-9 and ICD-10 codes. (PDF) [file pone.0099906.s002.pdf]

Table S2. Acute alcohol-related causes by AAF and their corresponding ICD-9 and ICD-10 codes.

Cause	ICD-9	ICD-10
100% Attributable		
Alcohol poisoning	980.0, 980.1, E860.0, E860.1, E860.2, E860.9	X45, Y15, T51.0, T51.1, T51.9
Suicide by and exposure to alcohol	Nil	X65
Excessive blood level of alcohol	790.3	R78.0
Direct Alcohol-Attributable Fractions Estimates		
Air-space transport	E840-E845	V95-V97
Aspiration	E911	W78-W79
Child maltreatment	E960-E968	X85-Y09, Y87.1
Drowning injuries	E910	W65-W74
Fall injuries	E880-E888, E848	W00-W19
Fire injuries	E890-E899	X00-X09
Firearms	E922	W32-W34
Homicide	E960-E969	X85-Y09, Y87.1
Hypothermia	E901	X31
Motor-vehicle nontraffic crashes	E820-E825	V02.0, V03.0, V04.0, V09.0, V12-V14(.0-.2), V19.0-V19.3, V20-V28(.0-.2), V29.0-V29.3, V30-V39(.0-.3),V40-V49(.0-.3), V50-V59(.0-.3), V60-V69(.0-.3), V70-V79(.0-.3), V81.0, V82.0, V83-V86(.4-.9), V88.0-V88.8, V89.0
Motor-vehicle traffic crashes	E810-E819	V02(.1, .9), V03(.1, .9), V04(.1, .9), V09.2, V12-V14(.3-.9), V19.4-V19.6, V20-V28(.3-.9), V29.4-V29.9, V30-V39(.4-.9), V40-V49(.4-.9), V50-V59(.4-.9), V60-V69(.4-.9), V70-V79(.4-.9), V80.3-V80.5, V81.1, V82.1, V83-V86(.0-.3), V87.0-V87.8, V89.2
Occupational and machine injuries	E917-E920	W24-W31, W45
Other road vehicle crashes	E800-E807, E826-E829	V01, V05-V06, V09.1, V09.3, V09.9, V10-V11, V15-V18, V19.3, V19.8-V19.9, V80.0-V80.2, V80.6-V80.9, V81.2-V81.9, V82.2-V82.9, V87.9, V88.9, V89.1, V89.3, V89.9
Poisoning (not alcohol)	E850-E869, E924.1	X40-X49 (except X45)
Suicide	E950-E959	X60-X84, (except X65) Y87.0
Water transport	E830-E838	V90-V94
