# Supplementary material for: Alcohol Tax Policy and Related Mortality. An Age-Period-Cohort Analysis of a Rapidly Developed Chinese Population, 1981–2010
Source: PLoS One. 2014 Aug 25;9(8):e99906. doi: 10.1371/journal.pone.0099906 (PMC4143164; doi:10.1371/journal.pone.0099906)
Supplement: Note S1 — Technical Appendix of the Poisson age-period-cohort model. (PDF) [file pone.0099906.s003.pdf]

Note S1. Technical Appendix of the Poisson age-period-cohort model

Sex-specific Poisson regression was used to estimate the relative risks (with 95% confidence intervals) of alcohol-related mortality by age, calendar period, and birth cohort. I assumed that the number of observed mortality cases, c_{ij}, out of a population size of n_{ij} women in age group i in time period j, followed a Poisson distribution with mean μ_{ij}. Under the full age-period-cohort model, the mean is specified as:

$$\log(\mu_{ij}) = \alpha_{age_i} + \beta_{period_j} + \gamma_{cohort_k} + \log(n_{ij}),$$

where α_{age_i} $(i = 1, \ldots, I)$ is the age effect, β_{period_j} is the period effect $(j = 1, \cdots, J)$, γ_{cohort_k} is the cohort effect $(k = 1, \ldots, K,\ k = I + j - i)$, and $\log(n_{ij})$ is the offset term.
